# Development and External Validation of a Nomogram for Predicting Cancer-Specific Survival of Non-Small Cell Lung Cancer Patients With Ipsilateral Pleural Dissemination

**DOI:** 10.3389/fonc.2021.645486

**Published:** 2021-07-19

**Authors:** Zhenfan Wang, Hao Li, Taorui Liu, Zewen Sun, Fan Yang, Guanchao Jiang

**Affiliations:** Department of Thoracic Surgery, Centre of Thoracic Minimally Invasive Surgery, Peking University People’s Hospital, Beijing, China

**Keywords:** nomogram, cancer-specific survival, non-small cell lung cancer, ipsilateral pleural dissemination, surgery

## Abstract

**Background:**

Non-small-cell lung cancer (NSCLC) patients with ipsilateral pleural dissemination are defined as M1a in the eighth of American Joint Committee on Cancer (AJCC) TNM staging. We aimed to build a nomogram to predict lung cancer specific survival (LCSS) of NSCLC patients with ipsilateral pleural dissemination and to compare the impact of primary tumor resection (PTR) on LCSS among patients with different features.

**Methods:**

A total of 3,918 NSCLC patients with ipsilateral pleural dissemination were identified from the Surveillance, Epidemiology, and End Results (SEER) database. We selected and integrated significant prognostic factors based on competing risk regression to build a nomogram. The model was subjected to internal validation within SEER cohort and external validation with the cohort of 97 patients from Peking University People’s Hospital.

**Results:**

Age (*P* < 0.001), gender (*P* = 0.037), T stage (*P* = 0.002), N stage (*P* < 0.001), metastasis pattern (*P* = 0.005), chemotherapy (*P* < 0.001), and PTR (*P* < 0.001) were independent prognostic factors. The calibration curves presented a good consistency and the Harrell’s C-index of nomogram were 0.682 (95%CI: 0.673–0.691), 0.687 (95%CI: 0.670–0.704) and 0.667 (95%CI: 0.584–0.750) in training, internal, and external validation cohort, respectively. Interaction tests suggested a greater LCSS difference caused by PTR in patients without chemotherapy (P < 0.001).

**Conclusions:**

We developed a nomogram based on competing risk regression to reliably predict prognosis of NSCLC patients with ipsilateral pleural dissemination and validated this nomogram in an external Chinese cohort. This novel nomogram might be a practical tool for clinicians to anticipate the 1-, 3- and 5-year LCSS for NSCLC patients with pleural dissemination. Subgroup analysis indicated that patients without chemotherapy could get more benefit from PTR. In order to assess the role of PTR in the management of M1a patients more accurately, further prospective study would be urgently required.

## Introduction

Lung cancer has the highest mortality rate worldwide despite advances in diagnostic and therapeutic techniques. More than one-third of non-small-cell lung cancers (NSCLCs) are diagnosed at stage IV of the disease (1). In the 7th edition of tumor, node and metastasis (TNM) lung cancer staging system, stage IV patients were subdivided and a new M descriptors of M1a were proposed, which was defined as patients with metastasis in the chest cavity, including malignant pleural effusion/nodules, pericardial effusion and contralateral pulmonary nodules (2). According to the International Association for the Study of Lung Cancer (IASLC) staging project, the median survival time (MST) and 5-year survival rate of these M1a patients were 8–11.5 months and 2–10% (2, 3), respectively. In 2017, M1a patients were subdivided as stage IVA in the 8th edition of TNM staging system (3).

Currently, research on the diagnosis and management of NSCLC patients with malignant contralateral pulmonary nodules has generally come to a fundamental consensus (4); however, it is more complicated and controversial for the treatment of patients with ipsilateral pleural dissemination, including malignant pleural effusion/nodules, pericardial effusion. Several studies have focused on the survival of these patients. Dai et al. (5) reported that lymph node involvement was an independent prognostic factor for lung cancer specific survival (LCSS) among all M1a patients, and Wang et al. (6) showed a similar result in patients with unexpected pleural spread at thoracotomy. Our previous study has demonstrated that primary tumor resection (PTR) brought favorable impact on both overall survival (OS) and LCSS for patients with ipsilateral pleural dissemination (7), especially for non-targeted therapy patients (8). Nevertheless, a predictive model specifically describing the LCSS of patients with ipsilateral pleural dissemination is not yet available, and the question about which type of patients are more suitable for PTR remains unclear.

Therefore, we aimed to develop and validate a novel nomogram based on competing risk regression predict the LCSS of NSCLC patients with ipsilateral pleural dissemination and to compare the impact of PTR on LCSS among patients with different feature.

## Method

### Study Population and Selection Criteria

The SEER program, managed by the National Cancer Institute, is one of the largest public databases that collect cancer incidence data from population-based cancer registries covering approximately 30% of the U.S. population. We used the SEER*Stat software (version 8.3.6 https://seer.cancer.gov/data-software/ Surveillance Research Program, National Cancer Institute, Maryland, USA) to derive information of patients from the Incidence-SEER 18 Regs Custom Data (with additional treatment fields), Nov 2019 Submission. The inclusion criteria for patient selection in this study were (a) patients diagnosed with pathologically confirmed NSCLC between 2010 and 2015, (b) stage IV and M1a disease according to the 7th edition of the American Joint Committee on Cancer (AJCC) TNM classification, (c) the SEER variable ‘CS Mets at DX’ with codes 15, 20, and 24 for ipsilateral pleural effusion, pericardial effusion, and pleural nodules on the ipsilateral lung separated from direct invasion, (d) only one malignant primary lesion. The exclusion criteria included (a) patients younger than 18 years old at the time of diagnosis, (b) metastases in the contralateral lung, (c) data on the survival time, cause of death, surgery information, and tumor size were unavailable, (d) survival time was recorded as zero month.

The independent external validation cohort was derived from NSCLC patients treated in the Peking University People’s Hospital, Beijing, China between January 1, 2006 and December 31, 2016. The study time of the validation cohort was quite long to enroll more M1a patients as possible. The inclusion criteria included pathologically confirmed ipsilateral pleural dissemination, age of 18 years or older and complete follow-up information. Patients with malignant contralateral pulmonary nodules, distant organs metastases or history of other malignancies were excluded. Informed consent was waived for this retrospective study by the Research Ethics Committee of the Peking University People’s Hospital. Patients treated in our center were followed up every 3 months in the first year and every 6 months thereafter until death. Physical examination, chest computed tomographic (CT) scans and tests of blood tumor markers were conducted routinely at follow-up, and magnetic resonance imaging (MRI) of the brain or 18F-FDG PET–CT was performed if necessary.

### Study Variables

The following information for each patient was extracted: baseline sociodemographic information (age, race, gender, vital status, cause of death, and survival months), tumor characteristics (tumor size, anatomic site, histological subtype, T stage, N stage, differentiation grade, and metastasis pattern: pleural effusion, pericardial effusion or pleural nodules) and treatment information (surgery, chemotherapy and radiation). Pleural decortication, pleurodesis, intraoperative intrapleural hyperthermic perfusion or other intrapleural operations were not included because those procedures were not performed in our center and also not documented in the SEER database. In this study, the histological subtypes were classified as adenocarcinoma, squamous cell carcinoma, large cell carcinoma, and adenosquamous carcinoma. We adjusted the TNM stage of each patient according to the 8th AJCC TNM classification system. In the subgroup analysis, the cut-off value of age at diagnosis was set at median (<70 and ≥70) with reference to the cut-off points used in previous studies (9). Lung cancer-specific survival (LCSS) was the study endpoint, which was defined as the time from the diagnosis to death attributed to lung cancer-specific mortality (LCSM).

### Construction of the Nomogram

In this study, all eligible patients from the SEER database (n = 3,918) were randomly assigned into training (70%, n = 2,745) and validation cohort (30%, n = 1,173) to establish and validate the nomogram. This ratio (7:3) ensured the maximal utilization of the data for constructing predictive model with a considerable number of sample size for validation (10–15).

The baseline clinicopathological characteristics and treatment information were analyzed using descriptive methods, with standard summary statistics including median, interquartile range (IQR), and proportions. Differences for continuous, non-normally distributed data were processed by the Mann–Whitney test. Categorical variables were compared by chi-square or Fisher’s exact tests, as appropriate.

Fine–Gray competing risk regression was performed to estimate the subhazard ratio (SHR) and evaluate the ability of the parameter in predicting the risk of LCSM, with non-cancer deaths as the competing risk (16). Variables with *P*-value <0.10 identified in univariable analyses were enrolled into multivariable regression. A nomogram was developed based on the prognostic factors with *P*-value < 0.05 in the multivariable analyses.

### Validation of the Nomogram

The model was subjected to internal validation in the SEER training cohort, independent validation in the SEER validation cohort, and external validation with the cohort from Peking University People’s Hospital. The performance of our nomogram was evaluated by calibration curves (500 bootstrap resamples), Harrell’s concordance index (C-index) (17), and the time-dependent receiver operating characteristic curve (the time-dependent ROC curve) (18). The calibration curves were depicted on the basis of predicted and observed probabilities of LCSM, which represented the calibration of our model. Discrimination ability was reflected by C-index and the area under the curves (AUCs) of ROC curves for 1-, 3- and 5-year LCSS, with values closer to 1.0 denoting better discrimination ability.

### Modified Nomogram and Subgroup Analysis

To perform an exploratory analysis about the impact of PTR on the LCSS among patients with different risks, we built a modified nomogram including the independent prognostic factors except for surgery status using training cohort. All cases were divided into low-risk and high-risk groups using the cut-off set at the highest third of the risk score calculated from the modified nomogram among training cohort, and the cumulative incidence of LCSM curves in different groups was delineated. Subgroup analysis stratified by clinicopathologic feature based on Fine–Gray test was conducted to compare the influence of PTR on LCSS within each subgroup.

Data analysis were performed using Stata/SE 15.0 for Windows (StataCorp, College Station, TX) and R software version 3.6.0. We used the “mstate” and “rms” package in R software to construct the nomogram, “pec” package to evaluate our model, and “nomogramEx” package to calculate the total score based on nomogram. All statistical tests were two-tailed, and a *P*-value less than 0.05 was considered statistically significant.

## Result

### Patient Characteristics

A total of 3,918 NSCLC patients with ipsilateral pleural dissemination from the SEER database and 97 eligible patients from Peking University People’s Hospital were included in this study. The baseline clinicopathological characteristics and treatment information are shown in **Table 1**. All eligible cases from the SEER database (n = 3,918) were randomly divided into training (70%, n = 2,745) and internal validation cohorts (30%, n = 1,173) to develop and validate the nomogram. The patient characteristics were comparable between these two cohorts (all parameters *P*-value >0.05). During a median follow-up times of 8 months (IQR: 3–18), 9 months (IQR: 3–18), and 32 months (IQR: 22–47), 2,145 (78.1%), 941 (80.2%), and 53 (54.6%) LCSMs were recorded in the training cohort, internal validation cohort, and external validation cohort, respectively. The difference of follow-up time between the two cohorts was mainly because near half of the SEER cohort (n = 1,922, 49.1%) died within the 8 months of follow-up, shortening the overall follow-up time of the SEER cohort.

**Table 1:** Baseline clinicopathological characteristics and treatment information of all, training, and validation cohorts.

	Training cohort	Internal validation cohort	External validation cohort
Number of cases	2745	1173	97
Age, years, median (IQR)	70 (61–78)	69 (61–77)	58 (50–67)
Race, n (%)			
White	2069 (75.4)	860 (73.3)	—
Black	399 (14.5)	179 (15.3)	—
Other	277 (10.1)	134 (11.4)	—
Gender, n (%)			
Male	1483 (54.0)	635 (54.1)	45 (46.4)
Female	1262 (46.0)	538 (45.9)	52 (53.6)
Anatomic sites, n (%)			
Bronchus	178 (6.5)	76 (6.5)	0 (0.0)
Lobe	2338 (85.2)	995 (84.9)	97 (100.0)
Unknown	229 (8.3)	102 (8.7)	0 (0)
Histological subtype, n (%)			
Adenocarcinoma	1831 (66.7)	794 (67.7)	91 (93.8)
Squamous cell carcinoma	827 (30.1)	343 (29.2)	5 (5.2)
Large cell carcinoma	42 (1.5)	14 (1.2)	0
Adenosquamous carcinoma	45 (1.6)	22 (1.9)	1 (1.0)
Tumor size, mm, median (IQR)	45 (28–67)	43 (28–66)	27 (20–42)
T stage, n (%)			
T1	251 (9.1)	120 (10.2)	35 (36.1)
T2	627 (22.8)	273 (23.3)	28 (28.9)
T3	640 (23.3)	260 (22.2)	9 (9.3)
T4	1227 (44.7)	520 (44.3)	25 (25.8)
N stage, n (%)			
N0	877 (32.0)	365 (31.1)	78 (80.4)
N1	217 (7.9)	94 (8.0)	3 (3.1)
N2	1291 (47.0)	567 (48.3)	16 (16.5)
N3	360 (13.1)	147 (12.6)	0 (0)
Metastasis pattern, n (%)			
Pleural nodules	443 (16.1)	202 (17.2)	53 (54.6)
Pleural effusion	1999 (72.8)	858 (73.2)	42 (43.3)
Pericardial effusion	303 (11.1)	113 (9.6)	2 (2.1)
Chemotherapy ^a^, n (%)			
Yes	1704 (62.1)	733 (62.5)	53 (54.6)
No	1041 (37.9)	440 (37.5)	44 (45.4)
Radiotherapy ^a^, n (%)			
Yes	173 (6.3)	85 (7.3)	7 (7.2)
No	2572 (93.7)	1088 (92.7)	90 (92.8)
Primary tumor resection, n (%)			
Yes	167 (6.1)	87 (7.4)	51 (52.6)
No	2578 (93.9)	1086 (92.6)	46 (47.4)
Extent of surgery, n (%)			
Local tumor destruction	8 (4.8)	3 (3.4)	0 (0)
Sublobar resection	70 (41.9)	37 (42.5)	39 (76.5)
(Bi)lobectomy ^b^	66 (39.5)	40 (46.0)	11 (21.5)
Pneumonectomy	23 (13.8)	7 (8.1)	1 (2.0)

IQR, interquartile range.

^a^These factors do not distinguish between before and after surgery.

^b^Includes lobectomy and bilobectomy.

The proportion of patient who underwent PTR in the external validation cohort was relatively higher than that in the training cohort, since our department is a high-volume surgical center, and most of our patients underwent surgical intervention. We depicted two cumulative incidence curves to compare the LCSS between patients with PTR in the SEER and our external cohort. We found that the patients who received PTR in the external validation cohort showed a significant better LCSS than patients in the SEER cohort (**Supplementary Figure 1**). The main reason might be that the patients treated in our department were highly selected. The different baseline features between training and external validation cohorts ensured an effective test for the generalization ability of our model.

In the external validation cohort, 52 patients received adjuvant chemotherapy only, one patient underwent neoadjuvant chemotherapy only and one patient received both adjuvant chemotherapy and neoadjuvant chemotherapy. Our external validation cohort documented the information of target therapy, which was lacking in the SEER database. Fifty-two patients received targeted therapy in the external validation cohort, and they showed better survival than patients without targeted therapy (MST: 57 *vs* 26 months, *P* < 0.001).

### Independent Prognostic Factors in the Training Cohort

The results of univariable and multivariable analyses were described in **Table 2**. Considering the lack of direct link between race and LCSS and the fact that variable T stage contains the information of tumor size, we did not include race and tumor size in the regression analysis. The univariable analysis indicated that age (*P* < 0.001), gender (*P* = 0.009), histological subtype (*P* = 0.033), T stage (*P* = 0.001), N stage (*P* = 0.003), metastasis pattern (*P* < 0.001), chemotherapy (*P* < 0.001), and PTR (*P* < 0.001) were significant prognostic factors. All significant prognostic factors were entered into the multivariable analysis based on competing risk regression, which revealed age (P < 0.001), gender (*P* = 0.037), T stage (*P* = 0.002), N stage (*P* < 0.001), metastasis pattern (*P* = 0.005), chemotherapy (*P* < 0.001), and PTR (*P* < 0.001) were independent prognostic factors to predict LCSS.

**Table 2:** Univariable and multivariable analyses of the ability of each factor in predicting LCSS in the training cohort.

	Univariable analysis		Multivariable predictors	
	SHR (95% CI)	*P*-value	SHR (95% CI)	*P*-value
Age	1.014 (1.011–1.018)	**<0.001**	1.008 (1.004–1.012)	**<0.001**
Gender		**0.009**		
Male	Reference		Reference	
Female	0.896 (0.825–0.973)		0.911 (0.835–0.994)	**0.037**
Anatomic sites		0.554		
Bronchus	Reference			
Lobe	0.912 90.763–1.091)			
Unknown	0.946 (0.756–1.185)			
Histological		**0.033**		
Adenocarcinoma	Reference			
Squamous cell carcinoma	1.143 (1.043–1.253)		0.986 (0.891–1.090)	0.780
Large cell carcinoma	0.927 (0.658–1.305)		0.814 (0.580–1.142)	0.234
Adenosquamous carcinoma	0.973 (0.689–1.374)		0.935 (0.672–1.300)	0.691
T stage		**0.001**		
T1	Reference		Reference	
T2	1.159 (0.980–1.371)		1.154 (0.971–1.372)	0.104
T3	1.186 (1.003–1.402)		1.166 (0.980–1.387)	0.084
T4	1.329 (1.134–1.558)		1.332 (1.127–1.575)	**0.001**
N stage		**0.003**		
N0	Reference		Reference	
N1	1.003 (0.850–1.183)		1.134 (0.956–1.344)	0.149
N2	1.153 (1.049–1.268)		1.246 (1.124–1.383)	<0.001
N3	1.214 (1.067–1.382)		1.371 (1.191–1.579)	<0.001
Metastasis pattern		**<0.001**		
Pleural nodules	Reference		Reference	
Pleural effusion	1.324 (1.184–1.479)		1.120 (1.072–1.354)	**0.002**
Pericardial effusion	1.380 (1.168–1.630)		1.242 (1.042–1.481)	**0.016**
Chemotherapy		**<0.001**		
No	Reference		Reference	
Yes	0.558 (0.510–0.611)		0.565 (0.512–0.624)	**<0.001**
Radiotherapy		0.137		
No	Reference			
Yes	0.877 (0.737–1.043)			
Primary tumor resection		**<0.001**		
No	Reference		Reference	
Yes	0.527 (0.434–0.637)		0.580 (0.476–0.708)	**<0.001**

The significant P value is in bold font.

### Constructing and Validating the Prognostic Nomogram

**Figure 1:**
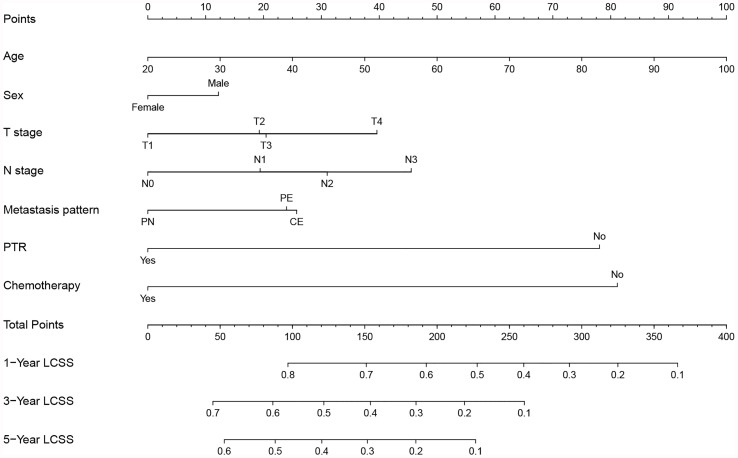
Prognostic nomogram predicting probability of 1-, 3- and 5-year lung cancer-specific survival (LCSS) in NSCLC patients with ipsilateral pleural dissemination. PN, pleural nodules; PE, pleural effusion; CE, pericardial effusion; PTR, primary tumor resection.

All independent prognostic factors mentioned above were incorporated to build the predictive model, which was visualized in the form of a nomogram (**Figure 1**). The nomogram illustrated that age was the most predominant contributor to the LCSS followed by surgical treatment and chemotherapy. T stage and N stage merely showed a moderate impact on LCSS. Each subtype of the predictors was assigned a score, ranging from 0 (lowest risk) to 100 (highest risk). The estimated probability of LCSS can be easily obtained by drawing a vertical line through the location of total score at the bottom scale.

**Figure 2:**
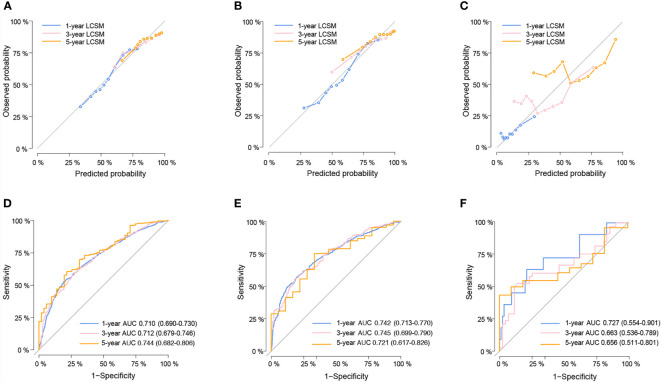
The calibration curves for predicting lung cancer-specific survival (LCSS) in the training **(A)**, internal validation **(B)** and external validation cohort **(C)** respectively. Nomogram-predicted probability is plotted on the x-axis; actual probability is plotted on the y-axis. A curve along the 45-degree line indicates perfect calibration models. Time-dependent ROC curves for the 1-, 3-, and 5-year LCSS probability in the training **(D)**, internal validation **(E)** and external validation cohorts **(F)** respectively.

The validation of the nomogram was shown in **Figure 2**. The calibration curves presented a good consistency between the nomogram predicted and actually observed 1-, 3-, and 5-year LCSS in the training, internal, and external validation cohorts (**Figures 2A–C**). The Harrell’s C-indices of nomogram were 0.682 (95%CI: 0.673–0.691), 0.687 (95%CI: 0.670–0.704), and 0.667 (95%CI: 0.584–0.750) in the training, internal, and external validation cohorts, respectively. The AUCs of 1-, 3-, and 5-year ROC also suggested a great predictive power for LCSS at these three timepoints (**Figures 2D–F**).

### Impact of PTR on Patients With Different Risk and Subgroup Analysis

**Figure 3:**
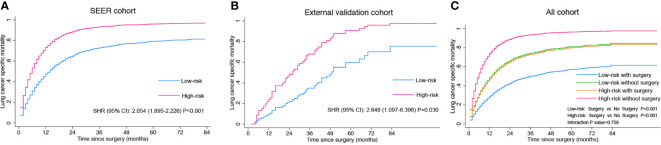
Risk group stratification according to the modified nomogram in the SEER cohort **(A)**, external validation cohort **(B)** and all cohorts **(C)**.

**Figure 4:**
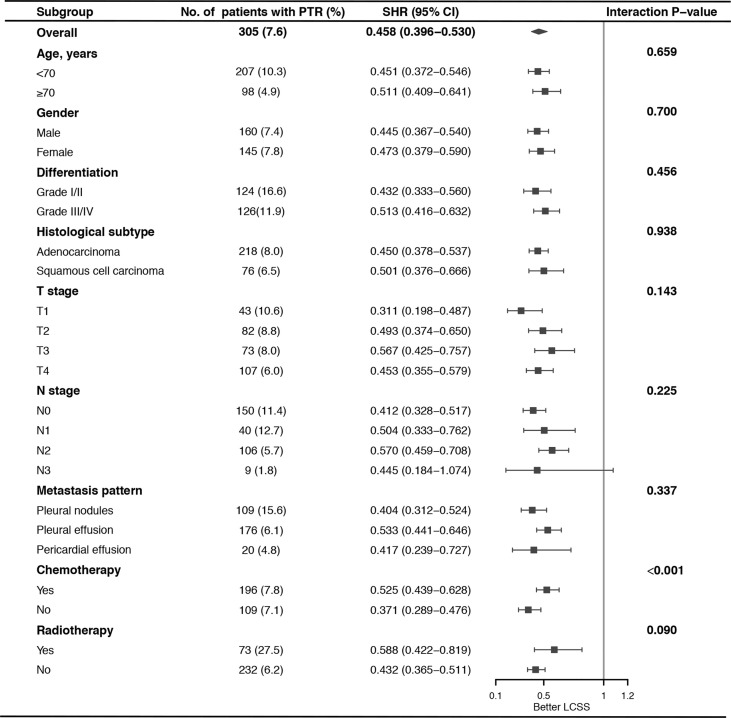
Subgroup analysis of the impact of primary tumor resection (PTR) on lung cancer-specific survival (LCSS) stratified by clinicopathologic feature.

To elucidate the impact of PTR on LCSS among patients with different risks, we established a modified nomogram including age, gender, T stage, N stage, metastasis pattern, and chemotherapy (**Supplementary Figure 1**) using the training cohort and calculated the risk score for LCSM of each case based on this modified nomogram. All the patients were classified into low-risk (n = 2,706, 67.4%, score <176.8) and high-risk group (n = 1,309, 32.6%, score≥176.8) with the cut-off point set at the highest third of the score in training cohort. Specifically, 260 (9.6%) low risk patients and 45 (3.4%) high risk patients underwent PTR. These two risk groups showed distinct cumulative incidence curves of LCSM in both SEER and external validation cohorts (**Figures 3A, B**), and surgery experience made significant favorable impact on LCSM within each risk group (**Figure 3C**). Moreover, there was no significant interaction effect between PTR and risk group, indicating similar benefit of PTR can be reaped by patients in different risk groups. Subgroup analysis and interaction tests were performed among all cases to identified patients who might get more benefit from PTR. We found that PTR was the favorable predictor of LCSS in almost all subgroups. Interestingly, interaction tests suggested a greater LCSS difference caused by PTR in patients without chemotherapy than in patients who received chemotherapy (**Figure 4**).

## Discussion

NSCLC with ipsilateral pleural dissemination, which was previously classified as T4 stage (19), is considered as M1a disease in the current 8th TNM staging system due to the extremely poor survival outcomes (3). Currently, prognostic prediction model for these cases is unavailable, and which type of patients could benefit more from PTR is still unclear. Here, we used a large cohort from the SEER database to establish a nomogram predicting the LCSS of NSCLC patients with ipsilateral pleural dissemination. The external validation favored the satisfactory performance of the nomogram in prognostic prediction. We identified age, gender, TN stage, metastasis pattern, PTR, and chemotherapy as the independent factors for LCSS. We confirmed that surgery intervention was a significant favorable predictor of LCSS in almost all subgroups, except for patients with N3 disease, may be due to the small sample size of N3 patients who underwent surgery (n = 9). Furthermore, interaction tests indicated that patients without chemotherapy could get more benefit from PTR.

NSCLC with ipsilateral pleural dissemination was traditionally regarded as a contraindication for surgical intervention, and system chemotherapy was considered as standard treatment (20). However, more and more evidence has revealed the positive role of surgery in prolonging the survival of M1a patients. In 2001, Ichinose et al. (21) first reported an unexpectedly good survival outcome in patients with carcinomatous pleuritis of minimal disease who underwent resection of the primary tumor, with 5-year survival of 22.8%. Similarly, promising prognoses were observed among patients with pleural dissemination who underwent primary lesion resection in subsequent years of research (6, 22–28). However, most of these studies were single-center focused on the patients with pleural dissemination first detected during operation, which were a highly selected population. Thus, to reflect the real-world situation more precisely, we used large cohort from the SEER database in our previous study (7) that also indicated PTR was associated with better OS and LCSS in patients with ipsilateral pleural dissemination. In this study, we further explored impact of PTR on different subgroups. The survival benefit of PTR tended to be greater in lower T stage and N stage population, similar to the published literature (5, 6, 9, 26, 29, 30), although the interaction test fell short of statistical significance. It was firstly observed in our study that patients without chemotherapy could get more survival benefit from PTR, providing valuable thought for surgical decision making.

In clinical practice, lymph node metastasis is an essential demarcation criterion for the staging of M0 patients, whereas M1a patients are categorized as stage IV regardless of any N status (3), which means the impact of lymph node metastasis on M1a patients deserves more comprehensive studies. Iida et al. (9) used a Japanese multicenter prospective cohort to conduct a retrospective study including 329 patients with pleural carcinomatosis and reported that the best stage N status (N0/N1) was associated with significantly longer survival when compared with N2/N3. Dai et al. performed a retrospective study using a SEER cohort and found that lymph node metastasis was a significant prognostic factor for NSCLC patients with pleural dissemination, and they proposed a speculative explanation that patients with N0 disease might have minor malignant pleural effusion or localized pleural nodules, which could be effectively controlled by comprehensive treatment (5). Hu et al. (31) investigated the SEER database and found that lymph node metastasis was the independent factor with poor prognosis for NSCLC patients with malignant pericardial effusion. It was also confirmed that higher T stage was related with worse prognosis (6, 26).

Systemic chemotherapy was considered as the standard therapy for patients with ipsilateral pleural dissemination (20). Kimura et al. (32) reported that platinum-based chemotherapy may improve the clinical outcomes of patients with pleural dissemination. Here, we confirmed the favorable role of chemotherapy in M1a patients, and we further found PTR brought more survival benefit in non-chemotherapy patients. Our previous study found targeted therapy could significantly improve the OS of M1a patients, and PTR brought more benefit to patients without targeted therapy (8). We speculated that systematic treatment, such as chemotherapy and targeted therapy, could effectively reduce the tumor burden, which solely depended on surgical intervention in patients without systematic treatment. These results indicated PTR was a more valuable treatment for M1a patients who cannot undergo chemotherapy and targeted therapy. Notably, PTR had significant favorable impact on both patients receiving chemotherapy and patients without chemotherapy; therefore, when adjuvant chemotherapy was uncertain, PTR was still preferred for M1a patients. In fact, it is hard to guide surgical decision on the basis of chemotherapy strategy due to the fact that chemotherapy strategy is usually defined only once a thorough pathological analysis has been completed, usually several days after surgery.

Recently, several predictive models for patients with malignant pleural effusion or malignant pleural pericardial effusion had been published. Most of them focused on the concentration of biomarkers in the pleural effusion or serum (33–35); however, the higher cost and the variation of result owing to the different techniques (36), as well as the difficulty in collecting extra tissues in patients with poor physical condition, limited the application of these predictive models (37). Tian et al. used the data of NSCLC patients with malignant pleural effusion or pericardial effusion from SEER database between 2010 and 2015 to developed a nomogram, which included age, gender, race, primary site, histology type, TN status, and effusion patterns with a C-index of 0.736 (38). However, the authors did not excluded patients with contralateral pulmonary nodules or distant organ metastasis, and they did not ensure the patients they enrolled had only one malignant primary tumor. Our nomogram developed in this study had some advantages compared with previous nomogram. We only included patients with ipsilateral pleural dissemination, who were more controversial population in clinical practice. The use of competing risk model could effectively eliminate the influence of death competition on cancer-specific survival. The external validation in a Chinese cohort showed the reliability of the nomogram in predicting LCSS of patients with ipsilateral pleural dissemination. This novel nomogram might be a practical tool for clinicians to optimize the individual treatment strategy for patients with different risks.

This study also has some limitations. First, the SEER-based study was limited by its retrospective nature with inherent biases. Although we used multivariable competing risk regression analysis to control the impact of confounding covariates, the unavailable confounding factors could not be well ruled out, such as performance status, detailed histological and mutational features, surgical approach (open or VAST), and systematic therapy regimen (neoadjuvant or adjuvant chemotherapy) which were lacking in the SEER database. Second, we were not able to distinguish between clinical and pathological M1a staging due to the lack of information in the SEER database, which hindered further prognostic analysis. Third, our external validation cohort was relatively small compared with the SEER cohort due to the small proportion of M1a NSCLC patients in a surgical center. Further, multi-center cohort might be necessary for the external validation. Finally, we excluded patients whose survival time was recorded as zero month in the SEER cohort to exclude potential confounding factors, because these patients accounted for a large proportion of SEER cohort during the data process (approximately 14.2%). But it would also eliminate the influence of perioperative mortality on the survival analysis.

## Conclusion

We developed a nomogram based on competing risk regression to reliably predict prognosis of NSCLC patients with ipsilateral pleural dissemination and validated this nomogram in an external Chinese cohort. This novel nomogram might be a practical tool for clinicians to anticipate the 1-, 3- and 5-year LCSS for NSCLC patients with pleural dissemination. Subgroup analysis indicated that patients without chemotherapy could get more benefit from PTR. In order to assess the role of PTR in the management of M1a patients more accurately, further prospective study would be urgently required.

## Data Availability Statement

The raw data supporting the conclusions of this article will be made available by the authors, without undue reservation.

## Ethics Statement

Ethical review and approval was not required for the study on human participants in accordance with the local legislation and institutional requirements. The ethics committee waived the requirement of written informed consent for participation.

## Author Contributions

ZW and HL: data collection, data analysis, and drafting the article. TL and ZS: data collection. FY and GJ: conception of the work and critical revision of the article. GJ: approved the final version of the manuscript on behalf of all authors. All authors contributed to the article and approved the submitted version.

## Funding

This work was supported by Beijing Municipal Science and Technology Commission (Z181100001718190); the Major Research plan of National Natural Science of China (grant 92059203); the National Natural Science Foundation of China (grant 82002410 and 61877001); Peking University People’s Hospital Scientific Research Development Funds (grant RDY2020-02).

## Conflict of Interest

The authors declare that the research was conducted in the absence of any commercial or financial relationships that could be construed as a potential conflict of interest.
