# Supplementary material for: Development and External Validation of a Nomogram for Predicting Cancer-Specific Survival of Non-Small Cell Lung Cancer Patients With Ipsilateral Pleural Dissemination
Source: Front Oncol. 2021 Jul 19;11:645486. doi: 10.3389/fonc.2021.645486 (PMC8327084; doi:10.3389/fonc.2021.645486)
Supplement: Supplementary file 1 [file DataSheet_1.docx]

Supplementary Material


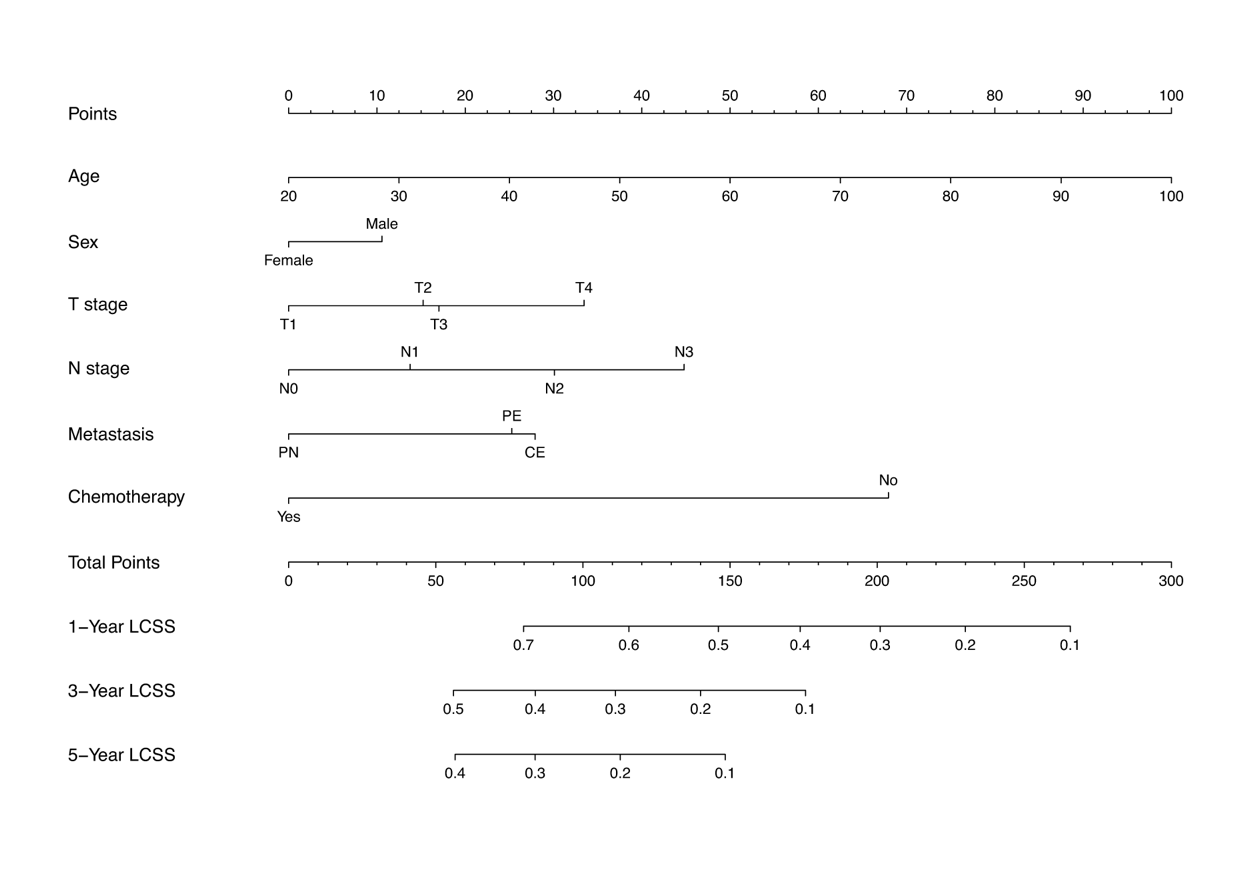


**Supplementary Figure 1.** A modified nomogram including age, gender, T stage, N stage, metastasis pattern and chemotherapy.
