# Supplementary figures and images for: Development and External Validation of a Nomogram for Predicting Cancer-Specific Survival of Non-Small Cell Lung Cancer Patients With Ipsilateral Pleural Dissemination
Source: Front Oncol. 2021 Jul 19;11:645486. doi: 10.3389/fonc.2021.645486 (PMC8327084; doi:10.3389/fonc.2021.645486)

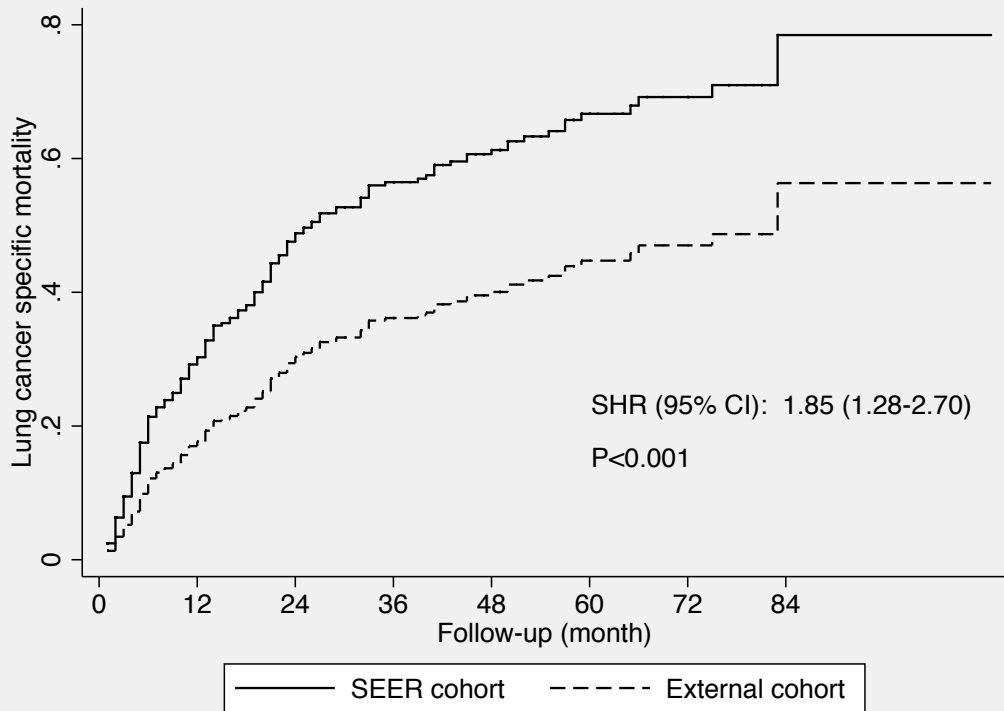

Supplement: Supplementary file 2 [file Image_1.pdf]
